# A further cost for the sicker sex? Evidence for male‐biased parasite‐induced vulnerability to predation

**DOI:** 10.1002/ece3.2049

**Published:** 2016-03-14

**Authors:** Jessica F. Stephenson, Cormac Kinsella, Joanne Cable, Cock van Oosterhout

**Affiliations:** ^1^ School of BiosciencesCardiff UniversityCardiffUK; ^2^ Center for Adaptation to a Changing Environment (ACE)ETH ZürichSwiss Federal Institute of TechnologyZürichSwitzerland; ^3^ Department of Aquatic EcologyEAWAGSwiss Federal Institute of Aquatic Science and TechnologyDübendorfSwitzerland; ^4^ School of Environmental SciencesUniversity of East AngliaNorwich Research ParkNorwichUK; ^5^ Present address: Evolutionary Biology CentreUppsala UniversityUppsalaSweden

**Keywords:** Bateman's principle for immunity, *Gyrodactylus*, infection tolerance, parasite‐induced vulnerability to predation, *Poecilia reticulata*, sex‐biased parasitism

## Abstract

Males are typically the sicker sex. Data from multiple taxa indicate that they are more likely to be infected with parasites, and are less “tolerant,” or less able to mitigate the fitness costs of a given infection, than females. One cost of infection for many animals is an increased probability of being captured by a predator. A clear, hitherto untested, prediction is therefore that this parasite‐induced vulnerability to predation is more pronounced among males than females. We tested this prediction in the sexually size dimorphic guppy, *Poecilia reticulata*, in which females are typically larger than males. We either sham or experimentally infected guppies with *Gyrodactylus turnbulli*, elicited their escape response using an established protocol and measured the distance they covered during 60 ms. To discriminate between the effects of body size and those of other inherent sex differences, we size‐matched fish across treatment groups. Infection with *G. turnbulli* reduced the distance covered during the escape response of small adults by 20.1%, whereas that of large fish was unaffected. This result implies that parasite‐induced vulnerability to predation is male‐biased in the wild: although there was no difference in escape response between our experimentally size‐matched groups of males and females, males are significantly smaller across natural guppy populations. These results are consistent with Bateman's principle for immunity: Natural selection for larger body sizes and longevity in females seems to have resulted in the evolution of increased infection tolerance. We discuss the potential implications of sex‐ and size‐biased parasite‐induced vulnerability to predation for the evolutionary ecology of this host–parasite interaction in natural communities.

## Introduction

Male animals are widely considered the sicker sex: Typically they are more likely to be infected with parasites, and less able to mitigate the fitness costs of a given infection (less “tolerant”; Råberg et al. 2009) than females across taxa (Poulin 1996; Zuk and McKean 1996). Although there are exceptions, a striking number of host–parasite interactions conform to this pattern (reviewed by Rolff 2002; Schmid‐Hempel 2011). Ultimately, this difference is due to sexual selection driving divergent life‐history strategies between the sexes (Bateman's principle; Bateman 1948; Rolff 2002). Males maximize fitness by mating with as many females as possible; they therefore invest more in male–male competition and reproduction, and generally have shorter life spans than females (Clutton‐Brock and Isvaran 2007; Zuk 2009). Females, by contrast, maximize fitness by lengthening life span and producing more broods; in order to do so they must invest more in self‐maintenance, such as defense against pathogens (Rolff 2002).

One intuitive and well‐established cost of parasite infection is that it increases the vulnerability of the host to predation. This effect could be due to manipulation by the parasite to facilitate trophic transmission, or simply a by‐product of the cost of infection (Johnson et al. 2010), and there is clear evidence of its importance from a variety of ecological systems. For example, parasite diversity and infection load reduce the ability of Iberian hares, *Lepus granatensis*, to evade capture (Alzaga et al. 2008), *Echinococcus granulosus* infection increases the vulnerability of moose, *Alces alces*, to predation by wolves, *Canis lupus* (see Joly and Messier 2004), and fish preferentially feed upon *Polycaryum laeve*‐infected *Daphnia* (see Johnson et al. 2006). To our knowledge, no previous study has tested whether the sexes differ in their ability to mitigate this cost of parasite infection.

The Trinidadian guppy, *Poecilia reticulata* Peters 1859, should provide an excellent example of female‐biased defense against infection. Females can use stored sperm to fertilize broods for up to 10 months after mating; males can therefore theoretically sire offspring long after death (Lopez‐Sepulcre et al. 2013). By contrast, females give birth to broods of live young, the sizes of which increase with maternal body size and, as female growth is indeterminate, age (Reznick et al. 1990). The reproductive fitness of female guppies is therefore intrinsically linked to longevity to a greater extent than that of males. Consequently, female life span is estimated to be 1.65 times longer than that of males (Arendt et al. 2014), which leads to generally female‐biased sex ratios in populations across rivers in Trinidad (Seghers 1974; McKellar et al. 2009; Arendt et al. 2014).

Recent research indicates a role for predators in driving patterns in the parasitism of natural guppy populations (Stephenson et al. 2015a,b), but the mechanisms involved remain unclear. The ectoparasitic monogeneans, *Gyrodactylus* spp. (at least three species: Xavier et al. 2015), are the most prevalent multicellular parasites in natural guppy populations (van Oosterhout et al. 2007; Stephenson et al. 2015b). Parasites in the *Gyrodactylus* genus feed on host mucous and epithelia and are transmitted directly through close contact between hosts (reviewed by Bakke et al. 2007). Field studies reveal that in populations experiencing high levels of predation pressure from aquatic predators such as the pike cichlid, *Crenicichla alta* (“high‐predation populations”; reviewed by Magurran 2005), infection with *Gyrodactylus* spp. is female‐biased (Gotanda et al. 2013; Stephenson et al. 2015b) or equal between the sexes (Martin and Johnsen 2007; Fraser et al. 2010), whereas those in the absence of major aquatic predators (“low‐predation populations”) show no sex bias (Martin and Johnsen 2007; Fraser et al. 2010; Stephenson et al. 2015b) or male‐biased *Gyrodactylus* spp. infection (Gotanda et al. 2013). One explanation of this pattern is that predator‐driven shoaling behavior, which facilitates parasite transmission, is more pronounced among females and thus drives female‐biased *Gyrodactylus* spp. infection in high‐predation populations (Stephenson et al. 2015b). An additional, nonmutually exclusive explanation is that parasite infection differentially affects the mortality of male and female guppies. Indeed, in natural populations, males appear to lose more body condition (a common measure of infection tolerance; Råberg et al. 2009) than females as a result of *Gyrodactylus* spp. infection (Stephenson et al. 2015a). A study by van Oosterhout et al. (2007) provides one source of differential mortality due to this difference in tolerance between the sexes: *Gyrodactylus* spp. infection makes male guppies, but not females, more likely to be swept downstream during flood events. Here, we investigate whether male‐biased *Gyrodactylus* spp.‐induced vulnerability to predation could be another.

In fish, an important component of predation avoidance is the escape response: a short (<1 sec), anaerobic burst of high‐velocity movement (Domenici and Blake 1997). Several studies have indicated that parasite infection can reduce fish escape responses (Barber et al. 2004; Östlund‐Nilsson et al. 2005; Blake et al. 2006; Binning et al. 2013), although the parasites involved are commonly so large that they alter fish morphology (e.g., *Schistocephalus solidus*, see Barber et al. 2004; Blake et al. 2006) or drag coefficient (e.g., isopods, Östlund‐Nilsson et al. 2005; Binning et al. 2013). It is likely, although previously untested, that the considerably smaller gyrodactylid worms (<1 mm) also reduce escape response: Previous studies report that guppies display reduced activity and swimming ability during *G. turnbulli* Harris 1986 infection and that this may be correlated with infection load (López 1998; van Oosterhout et al. 2003; Kolluru et al. 2009; Hockley et al. 2013). *G*. *turnbulli* is predominantly found on the tail and caudal peduncle (Harris 1988), where it reproduces rapidly on the host's skin with a generation time of less than 24 h at 25°C (Cable 2011). Because guppies vary in their ability to limit such parasite infrapopulation growth (Bush et al. 1997) (“resistance”: Råberg et al. 2009), even controlled experimental infections result in hosts with a range of infection loads, as can be directly observed on live fish (Scott and Anderson 1984). Here, we used a behavioral experiment to test whether *G. turnbulli* infection has a male‐biased effect on guppy escape response, and whether the effect is proportional to infection load. Although measuring the escape response is a proxy for predation risk, it is more ethically justifiable than exposing fish to live predators and is well documented as an important determinant of survival during such encounters (Domenici and Blake 1997; Bergstrom 2002; Ghalambor et al. 2004). With this caveat in mind, the results support the hypothesis that parasite‐induced vulnerability to predation could be male‐biased in natural populations.

## Materials and Methods

### Fish origin and maintenance

The guppies used to experimentally test the effect of infection with *Gyrodactylus turnbulli* on escape response were second‐generation laboratory‐bred descendants of wild guppies from a high‐predation population located at an undeveloped, rural site on the Caura River, Trinidad (UTM: 20 P 679527.7 m E, 1180376.4 m N based on WGS84 Datum; elevation 112 m). Wild guppies (n ~ 600) were transported to Cardiff University in June 2012 (Cefas APB authorization number CW054‐D‐187A), where they were prophylactically treated for infection using Binox^®^ (Nitrofurazone; Jungle Laboratories Corporation^®^, Cibolo, TX). This site was found *Gyrodactylus* spp. free in a previous survey (Stephenson et al. 2015b), and we failed to find any *Gyrodactylus* spp. during examination of a subset of 80 of the collected wild fish. Fish were housed in 70 L mixed sex stock tanks on a 12‐h light: 12‐h dark lighting regime (overhead fluorescent lighting) at 24 ± 1°C and fed daily with Aquarian^®^ tropical fish flakes supplemented with brine shrimp and bloodworm. Fry were removed from the breeding tanks soon after birth, confirmed free of ectoparasitic infection under a microscope, reared in one of four juvenile tanks until sex determination was possible (~6–8 weeks; males identified by the initiation of the modification of the anal fin, females by dark pigmentation at the base of the anal fin) and then reared in sex‐specific tanks for at least a further month before use. All fish used in the experiment were therefore sexually mature virgins, controlling for the fact that reproductive status influences the escape response of females (Ghalambor et al. 2004).

### Experimental treatments

**Figure 1:**
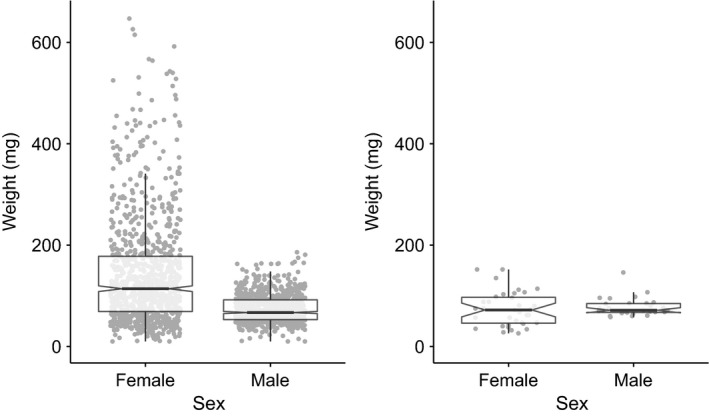
The left panel presents a notched box plot and raw data from field surveys and demonstrates that in populations experiencing high levels of predation pressure, such as the populations used in our behavioral experiment, female guppies are significantly larger than male guppies (*t*‐test: *t* (1767) = 16.94, *P *<* *0.0001). The collection of these data is described in Stephenson et al. (2015b). The right panel gives the equivalent data from our size‐matched experimental fish, which did not differ in size (*t*‐test: *t* (53.4) = −0.08, *P *=* *0.937).

We used 38 guppies (19 females, 19 males) as the “infected” treatment group and 30 guppies (19 females, 11 males) as the “uninfected” treatment group. These sample sizes were dictated by the number of fish available at the time of the experiment. We recorded the weight and standard length of all individuals immediately prior to infection. Across the populations of Trinidadian guppies sampled by Stephenson et al. (2015b), females were significantly larger than males (weight (mg) ± SEM females: 148.69 ± 2.71; males: 79.34 ± 0.75; *t‐*test: *t*(2016.4) = 24.64, *P* < 0.0001). Figure 1 illustrates the same pattern among populations experiencing high levels of predation pressure, such as the source population of our experimental fish. In order to discriminate between the effect of these inherently different sizes and other potential sex differences on escape response, we size‐matched males and females (Fig. 1; difference in mean weight: 0.5 mg; *t*‐test: *t* (66) = −0.07; *P* = 0.94), as well as the infected and uninfected fish (difference in mean weight: 7.8 mg; *t*‐test: *t* (66) = 0.98; *P* = 0.33).

Fish (overall mean standard length, mm ± SEM: 16.4 ± 0.3; mean weight, mg ± SEM: 73.4 ± 3.9) were held individually in 1 L containers and fed daily throughout the experiment. The water in these tanks was exchanged for clean dechlorinated water at least once every two days. We randomly assigned infected fish to batch 1 (12 females, eight males) or batch 2 (seven females, 11 males), allowing all behavioral trials to be conducted at an equivalent stage of infection (day 8: *n* = 10; or 9: *n* = 28; see Table S1 for further details). We used the *Gt3* strain of *G. turnbulli*, originally isolated from guppies in a pet shop in Nottingham, UK, in 1997. Fish were infected under anesthetic (0.02% tricaine methanesulfonate; MS222; PHARMAQ Ltd., Fordingbridge, UK) on day 0 through the transfer of 2.3 ± 0.1 (mean ± SEM) parasites from a heavily infected, recently killed donor, as observed using a dissecting microscope and fiber optic illumination. We monitored the infections under anesthetic (0.02% MS222) on days 1, 3, 5, 7, and 1 day post‐trial (day 9: *n* = 10; or 10: *n* = 28). Using this method, we also exposed the parasites to MS222. However, exposure was standardized between test fish, and parasites successfully established and reproduced on all test fish. Further, previous experiments suggest that the effect of such brief exposure to low doses of MS222 on *G. turnbulli* is negligible (JC, unpublished data).

### Experimental setup and design

**Figure 2:**
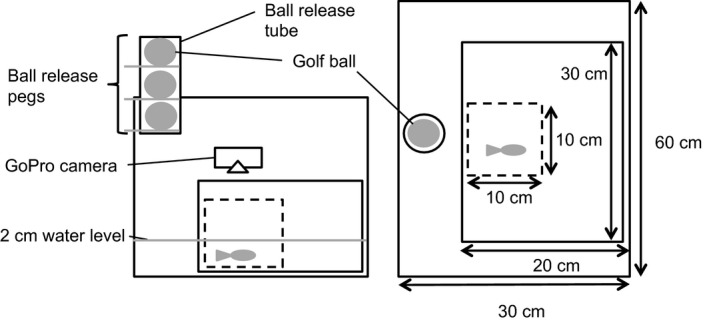
The apparatus used to test for the effect of infection with *Gyrodactylus turnbulli* on guppy escape response. The left panel gives a side view, the right an overhead view.

To assess their escape response, we individually exposed guppies to multiple startling stimuli within a trial arena. The experimental setup consisted of a 30 × 60 cm outer tank with a 20 × 30 cm inner tank, both filled with dechlorinated water at 25–26°C to a depth of 2 cm (Fig. 2). An arena constructed from plastic mesh (10 × 10 × 16 cm; Darice^®^ Inc., Strongsville, OH) was placed in the smaller tank on a white background. We selected the side length to approximately five body lengths of the test fish (Ghalambor et al. 2004). We situated a remotely operated ball release above the gap between the inner and outer tanks, which contained three golf balls at 35, 40, and 45 cm height, each with a separate release mechanism. No variation in the escape response was attributable to this difference in ball drop height (*P *>* *0.10, included as a factor in the general linear mixed models described below), which is unsurprising given that escape responses are a threshold “all‐or‐nothing” behavior (Bergstrom 2002).

At the start of each trial, a fish was placed in the arena and left for a 10‐min acclimatization period. The first ball was released after acclimatization, followed by the remaining two at 3‐min interval. A wirelessly operated camera (HERO3 + White Edition; GoPro Inc. San Mateo, CA), set to film at 60 frames per second, was suspended 20 cm above the arena and activated for approximately 15 sec during the release of each ball, so three videos were recorded per fish. Between the trials of individual fish, the inner tank was emptied and cleaned with 70% ethanol in order to remove any chemical cues or dislodged parasites. The 68 fish were tested over the course of 6 days. EthoVision XT (Noldus) was used to record the distance the fish travelled (mm) in the 60 ms immediately following the initiation of the escape response for each video (three videos per fish: *n* = 204). If multiple escape responses occurred following a single ball drop, only the first was analyzed.

### Ethical note

All animal work was conducted under the UK Home Office license (PPL 30/2876) with approval by the Cardiff University Animal Ethics Committee. During the course of this experiment, guppies were subjected to social isolation and some were additionally experimentally infected with parasites. Although this short‐term social isolation is likely to have temporarily elevated their stress levels, previous work using this method indicates that guppies show no sign of having suffered lasting harm (JFS, personal observation). To further mitigate potential effects of this isolation, fish were held in close proximity in transparent tanks and were in visual contact with conspecifics. Among the infected fish, none were infected for longer than 10 days. Infected fish were carefully monitored and none exhibited severe pathology at any point during the experiment (fused fin rays, difficulty swimming, loss of appetite; Cable 2011). Throughout the experiment, mortality was low (<5%) and infected fish recovered fully with treatment.

### Data analysis

We used two models to identify factors important in the effect of infection with *G. turnbulli* on the escape response of guppies. The first model used data from both infected and uninfected fish (Model 1), and the second used data from infected fish only (Model 2). For Model 2, we additionally removed the data from the three fish infected with over 100 worms, all male, so that the range and variance in infection load were the same between the sexes (see Fig. S1 for more information). Both models were general linear mixed models (Gaussian error family with an identity link function) in the *lme4* package in R (3.0.2; R Core Team 2013, Bates et al. 2015). For both models, we used the distance (mm) the fish moved in the 60 ms immediately following the initiation of the escape response as the response variable, and fish identity was included as a random effect to account for the three measurements taken per fish. Fixed effects included infection status (infected or uninfected; Model 1 only); infection load (how many *G. turnbulli* the fish was carrying when tested); fish weight (as a measure of size; see Table S2 for the results of Model 1 run with fish length as the size measure); fish sex; the position of the fish in the arena when the ball dropped (i.e., in the half nearest of furthest away from the ball); drop number (i.e., ball number 1, 2, or 3); and two‐ and three‐way interactions about which biologically relevant hypotheses had been made (see Table 1). We additionally included fish scaled mass index, a measure of condition, and its two‐way interactions with sex, infection load, and infection status (Model 1 only) as fixed effects in both models. Scaled mass index was calculated for each fish using the *lmodel2* R package (Legendre 2014) and the equation given by Peig and Green (2009), standardized such that both males and females had a mean SMI of 0 to account for differences in body shape between the sexes (Magurran 2005).

**Table 1:** The final model explaining variation in the distance all fish covered during 60 ms (Model 1). The starting model included the following factors: sex, infection status, weight, scaled mass index (and all two‐ and three‐way interactions between these three variables), infection load, position, drop number. Nonsignificant terms were removed sequentially to minimize AIC, and significant terms (at *α *= 0.05) in this final model are highlighted in bold

Parameter	Estimate	*F*	Degrees of freedom	*P*
Infection status (infected)	−9.90	4.25	1,55.4	**0.038**
Position (further from stimulus)	−5.36	21.02	1,144.9	**<0.0001**
Weight	−0.06	0.62	1, 49.4	0.439
Infection status × weight	0.09	5.67	1, 56.2	**0.020**

**Figure 3:**
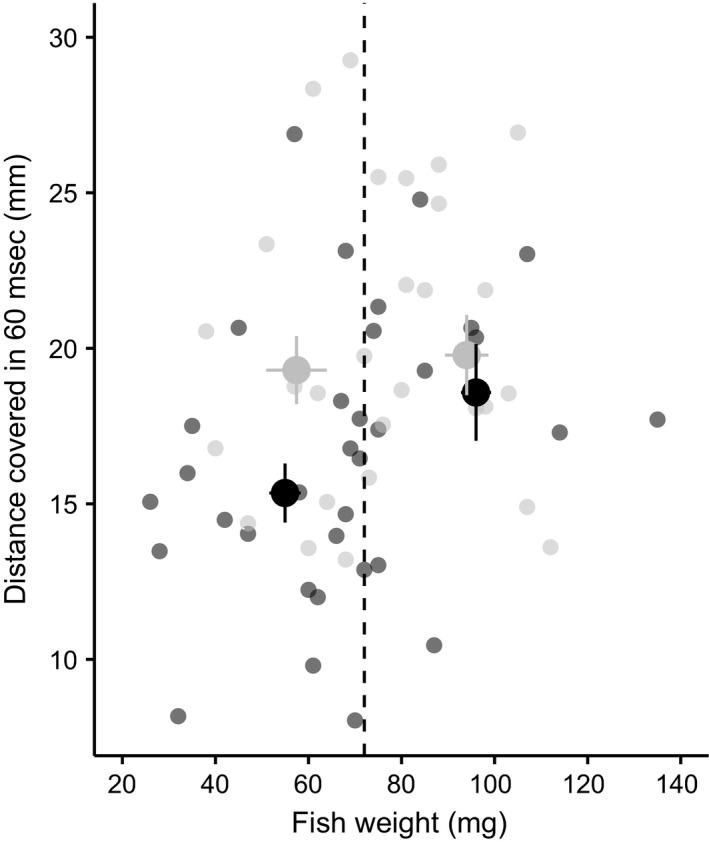
Infection with *Gyrodactylus turnbulli* reduced the distance that smaller fish covered during the escape response. There was no effect of infection on the escape response of large fish. Dark points are data from the infected fish, light from uninfected fish. The error bars are the standard error of the mean. The analysis was conducted using weight as a continuous variable, but for the means and standard errors in the figure, fish were divided into categories at the median value of 72 mg, delineated by the dashed line.

We confirmed that the residuals of both models conformed to the assumptions of normality and homogeneity of variance (Shapiro–Wilk tests; Model 1: W = 0.989, *P *=* *0.26; Model 2: W = 0.982; *P *=* *0.31; see Figs. S2–S5 for residual plots). We used weight as a continuous variable throughout the analyses, but for illustration purposes in Figure 3, we divided the fish into “small” and “large” categories at the median value of 72 mg. Both models were simplified by manually removing nonsignificant fixed effects in sequence from the full model, using likelihood ratio tests to confirm the validity of each step, to minimize Akaike's Information Criterion (AIC).

To test for potential effects of host sex, condition or size on the parasite infrapopulation growth rate, we calculated the integral of infection load over time for each recipient, which provides a single value encompassing both the duration and load of infection (Adelman et al. 2013). We used this integral value as the response variable in a general linear model in R (3.0.2; R Core Team 2013), and included fish weight, scaled mass index, sex, and their interactions as fixed factors.

## Results

Guppies infected with *Gyrodactylus turnbulli* moved on average 9.9 mm less during the escape response than uninfected guppies, equating to a reduction of ~37% (Table 1). However, how infection affected guppy escape response depended on fish size: for every mg in increased weight, infected guppies moved roughly 0.1 mm more during the escape response, whereas weight did not affect the escape response of uninfected fish (Fig. 3; infection status × fish weight interaction, Table 1). Similarly, for every 1 mm increase in length, infected fish moved 1.6 mm more during the escape response (infection status × fish length interaction, Table S2). For clarity, on Figure 3, we have also provided the mean values for fish above and below the median size of 72 mg. Analysis using these categories indicated that small infected fish covered an average of 20.1% less distance in the first 60 ms of the escape response than small uninfected fish, but infection did not affect the distance covered by large fish (Fig. 3). The random term of fish identity explained less than 0.1% of the variance, suggesting that responses within individual fish were as variable as those between individuals.

Among infected fish, the only factor that explained a significant amount of the variation in the response variable was the proximity of the fish to the ball when it dropped; intuitively, and as in Model 1, those closer moved furthest (*F*_1,74.1_ = 7.062; *P *=* *0.0096). Again, the random term of fish identity explained less than 0.1% of the variance.

There was no difference in the mean number of parasites carried by males and females over the course of their infection (mean number of parasites per fish ± SEM females: 16.48 ± 1.75; males: 20.73 ± 2.75; *t*‐test: *t* (36) = −1.29, *P *=* *0.20), nor at the time of testing (Fig. S1; females: 30.95 ± 5.58; males: 49.79 ± 9.96; *t*‐test: *t* (36) = −1.65, *P *=* *0.11). Additionally, the integral of infection load over time did not differ significantly between males and females nor was there a significant correlation with fish size, scaled mass index (condition), or an interaction between these terms (all *P *>* *0.3).

## Discussion

Infection with *Gyrodactylus turnbulli* reduced the distance guppies covered during the escape response, but the extent of this effect was dependent on the size of the fish (Fig. 3). Although this is a proxy measure, the reduction in the distance covered during the escape response among *G. turnbulli‐*infected fish indicates that these parasites increase vulnerability to predation, particularly of smaller fish (Domenici and Blake 1997; Bergstrom 2002; Ghalambor et al. 2004). As wild guppy females are significantly larger than males (Fig. 1), parasite‐induced vulnerability to predation is likely to be male‐biased in natural populations. This result predicts higher mortality among infected males than infected females in natural populations, a prediction supported by observational studies, which generally record female‐biased *Gyrodactylus* spp. infection in high‐predation populations (Gotanda et al. 2013; Stephenson et al. 2015b).

The mechanism by which infection with *G. turnbulli* reduced the distance guppies covered during the escape response is unclear. Host manipulation to facilitate trophic transmission is unlikely to contribute: *G. turnbulli* is unable to reproduce on hosts other than the guppy in the wild (Cable et al. 2013), and most likely dies during predation of its host. Parasites can affect fish swimming performance in a number of ways (reviewed by Barber et al. 2000). Previous studies show that infection with *Gyrodactylus* spp. causes reduced activity levels (van Oosterhout et al. 2003; Kolluru et al. 2009), and the pathology can include clamped fins (Cable et al. 2002) with obvious negative impacts on swimming (Hockley et al. 2013), particularly as *G. turnbulli* tends to aggregate on the tail fin (Harris 1988). While this effect could clearly be important, the infected fish in our experiment were not infected for long enough to develop such severe symptoms.

Although they had not developed severe symptoms, our experimental fish did have heavier infection loads than those generally observed in field surveys. We have observed infection loads of up to 272 in wild collections, with a mean ± SD among infected fish of 5.13 ± 9.90 in populations experiencing high levels of predation, such as our test population (Stephenson et al. 2015b). Among our experimental fish, the maximum infection load observed was 163, with a mean ± SD of 40.37 ± 36.02. We suggest two nonmutually exclusive explanations for this difference in mean infection load between wild and experimental fish. First, data suggest that infected fish are easier to catch, and therefore perhaps more often consumed than uninfected fish (present study), or more vulnerable to being swept downstream (van Oosterhout et al. 2007), so field collections may underestimate the *Gyrodactylus* spp. loads wild fish develop. Secondly, all of our experimental fish were near the peak of their infections at the point of testing, whereas fish collected from the field would have been sampled at a range of different time points of infection, which would naturally result in a lower mean infection load. Given that we still observed an effect of infection of the escape response, despite the fact that the experimental fish were maintained to the highest standards, fed *ad libitum*, and displayed no severe symptoms of infection, we feel confident that this effect is likely important among wild guppies.

The guppies we tested were at a stage of infection at which the immune response would have been activated (Bakke et al. 2007), and this itself may cause a reduction in escape response (Lochmiller and Deerenberg 2000): Stimulation of the immune response using nonpathogenic methods increases the vulnerability to predation of Eurasian collared doves (*Streptopelia decaocto*; see Eraud et al. 2009) and larval damselfly (*Coenagrion puella*; see Janssens and Stocks 2014). That we observed an effect of infection status, but not of infection load, suggests that immune activation may have had a more important effect on escape response than the parasite itself in this experiment.

The effect of infection on guppy escape response is size dependent: Smaller fish were less tolerant of infection than larger ones. Positive correlations between body size and parasite resistance or tolerance have been recorded in a number of species, for example, Soay sheep, *Ovis aries* (see Coltman et al. 2001) and garter snakes, *Thamnophis elegans* (see Sparkman and Palacios 2009). *Gyrodactylus* spp. infections are energetically costly (van Oosterhout et al. 2003; Bakke et al. 2007; Kolluru et al. 2009): Lower tolerance would likely manifest in physiological changes as a result of infection that could explain the decrease in escape response (Barber et al. 2000; Lochmiller and Deerenberg 2000). In support of this hypothesis, smaller guppies and males select habitats with lower velocity, less turbulent flow, particularly when infected with *G. turnbulli*, suggesting they are more energetically limited generally and are more affected by infection (Hockley et al. 2013). While previous work has indicated that smaller fish develop lower infection loads (van Oosterhout et al. 2008), the present study suggests that infection load is less important than the size of the fish in determining the effect of infection on escape response, and so perhaps fitness. Thus, despite generally developing heavier infection loads, larger fish may experience fewer negative effects of infection; parasitism could therefore be an important force in driving the evolution of larger body sizes in low‐predation populations of guppies.

We failed to find an inherent sex difference in the effect of infection on the escape response. The evidence for divergence in infection load and rate of parasite growth between the sexes is similarly inconclusive (e.g., van Oosterhout et al. 2003; Cable and van Oosterhout 2007; Pérez‐Jvostov et al. 2012). However, the divergence in life‐history strategies between the sexes does predict differences in their defense against parasites (Rolff 2002), and our recent study of wild guppies suggests a sex‐dependent effect of infection on body condition (Stephenson et al. 2015a). Study design may explain this discrepancy; experiments such as ours and those listed above expose parasite‐naïve fish to *Gyrodactylus* spp. Such experiments largely reveal differences in the innate immune response of previously unexposed fish, and it may be that sex differences occur only in the acquired immune response, with the longer‐lived females investing more, as suggested by interspecific comparisons (Previtali et al. 2012). Alternatively, males' ability to defend themselves against parasites may senesce faster than that of females: The guppies used in this and other studies may simply have been too young to reveal this divergence.

Despite the lack of an inherent sex effect in our data, the strong size‐dependent effect of infection on escape response coupled with the sexual size dimorphism in this species (Fig. 1) does suggest that in natural populations, parasite‐induced vulnerability to predation would be male‐biased. Such removal of infected males will have implications for the epidemiology of the disease in natural populations. Due to their low shoal fidelity and high contact rates resulting from the search for mating opportunities (Croft et al. 2003), males are likely to be key in both inter‐ and intrashoal transmission. Consequently, selective predation on infected males may reduce parasite prevalence and intensity in natural populations (Packer et al. 2003). It is also likely to exacerbate the male‐biased predation already implicated in driving female‐biased sex ratios in this species (McKellar et al. 2009).

**Figure 4:**
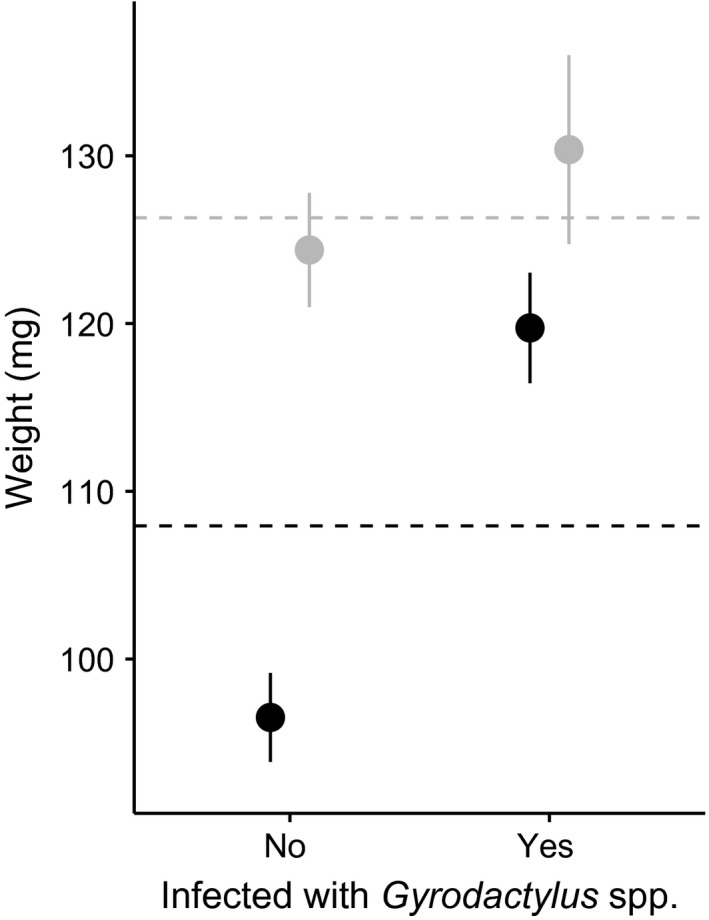
There is a clear size difference between *Gyrodactylus* spp.‐infected and uninfected guppies from lower course populations, which experience high levels of predation (black points), but not among guppies from upper course populations, which experience lower levels of predation (gray points). The dashed lines represent the mean weight of guppies from each predation regime, and the error bars give the standard error. These data come from the field collections described in Stephenson et al. (2015b).

We have provided evidence that the effects of predators on the distribution of parasites among host populations may be both direct (present study) and indirect (Stephenson et al. 2015a,b). It is clear from field data that both these processes are important. Figure 4 illustrates a pattern that has been observed by at least two independent field surveys (Fig. 2 in Stephenson et al. 2015b; Fig. 3 in Gotanda et al. 2013). In these surveys, relative to low‐predation populations, infected guppies smaller than the population mean size are underrepresented in high‐predation populations (Fig. 4). Based on the results of the present study, we propose that this pattern is due to the direct effect of predators: These small, high‐predation guppies are those most prone to parasite‐induced vulnerability to predation. Large uninfected guppies, again relative to the population mean size, are also scarce in high‐predation populations (Fig. 4). We have previously argued that this may be due to the indirect effects of predators: increased transmission due to increased shoaling results in higher re‐infection rates (Stephenson et al. 2015b). In summary, these findings clearly demonstrate that the community in which the host and parasite interact can have profound implications for that interaction and support recent calls for further investigation of the impact of community interactions, particularly predation, on disease ecology (Packer et al. 2003; Raffel et al. 2008).

## Data Accessibility

Raw data are available from Dryad Digital Repository. doi:10.5061/dryad.3n6s7

## Conflict of Interest

None declared.

## Supporting information


**Table S1**. Further information about the fish used during the trials on days 8 and 9 of infection.
**Table S2**. The final model explaining variation in the distance all fish covered during 60 ms.
**Figure S1**. The size and parasite loads of the fish used in the behavioural experiment, split by sex.
**Figure S2**. The histogram of the residuals from Model 1.
**Figure S3**. The plot of the residuals against fitted values from Model 1.
**Figure S4**. The histogram of the residuals from Model 2.
**Figure S5**. The residuals and fitted values from Model 2 (which included a single, binomial fixed effect).
