# Supplementary material for: A further cost for the sicker sex? Evidence for male‐biased parasite‐induced vulnerability to predation
Source: Ecol Evol. 2016 Mar 14;6(8):2506–15. doi: 10.1002/ece3.2049 (PMC4797162; doi:10.1002/ece3.2049)
Supplement: Supplementary file 1 — Table S1. Further information about the fish used during the trials on days 8 and 9 of infection. Table S2. The final model explaining variation in the distance all fish covered during 60 ms. Figure S1. The size and parasite loads of the fish used in the behavioural experiment, split by sex. Figure S2. The histogram of the residuals from Model 1. Figure S3. The plot of the residuals against fitted values from Model 1. Figure S4. The histogram of the residuals from Model 2. Figure S5. The residuals and fitted values from Model 2 (which included a single, binomial fixed effect). [file ECE3-6-2506-s001.docx]

**Supplementary Material:**

**A further cost for the sicker sex? Evidence for male-biased parasite-induced vulnerability to predation**

**Jessica F. Stephenson* ^a,b,c^, Cormac Kinsella^a,1^, Joanne Cable^a^, Cock van Oosterhout^b^**

^a^School of Biosciences, Cardiff University, Cardiff, UK

^b^Center for Adaptation to a Changing Environment (ACE), ETH Zürich, Swiss Federal Institute of Technology, Zürich, Switzerland

^c^Department of Aquatic Ecology, EAWAG, Swiss Federal Institute of Aquatic Science and Technology, Dübendorf, Switzerland

**^b^**School of Environmental Sciences, University of East Anglia, Norwich Research Park, Norwich, UK

^1^Present address: Evolutionary Biology Centre, Uppsala University, Uppsala, Sweden

*Correspondence: [jfrstephenson@gmail.com](mailto:jfrstephenson@gmail.com)

**Summary**

In this supplement we present extra tables and figures providing more information about the experimental design (Table S1), the data analyses (Table S2 and Figs S1-4), and the fish used in the experiment (Fig. S5).

| Day of infection | Number of infected fish  (Number of females) | Number of uninfected fish  (Number of females) | Mean±SE infection load of infected fish |
| --- | --- | --- | --- |
| 8 | 10 (6) | 18 (11) | 31.20±4.31 |
| 9 | 28 (13) | 12 (8) | 43.64±7.73 |

**Table S1** Further information about the fish used during the trials on days 8 and 9 of infection. We balanced the sex ratio, and the infection status of fish used at each of these time points. Although the infection loads of day 9 fish were higher than those of day 8 fish, the difference was not statistically significant (*t*-test: *t*(36)=-0.936; *P* = 0.355).

| Parameter | Estimate | *F* | Degrees of Freedom | *P* |
| --- | --- | --- | --- | --- |
| Infection status (infected) | -28.91 | 4.54 | 1, 56.13 | **0.038** |
| Position (further from stimulus) | -5.41 | 21.57 | 1, 146.0 | **<0.0001** |
| Length | -0.83 | 1.18 | 1, 49.60 | 0.441 |
| Infection status × length | 1.61 | 6.48 | 1, 56.33 | **0.014** |

**Table S2** The final model explaining variation in the distance all fish covered during 60 ms. The starting model included the following factors: sex; infection status; length; scaled mass index (and all two- and three-way interactions between these three variables); infection load; drop number. Non-significant terms were removed sequentially to minimise AIC, and significant terms (at α = 0.05) in this final model are highlighted in bold.

**Figure S1** The size and parasite loads of the fish used in the behavioural experiment, split by sex. Males (black and dark grey) and females (light grey) did not differ significantly in size or parasite load (statistics reported in the main text). The large points give the mean values and the error bars are the standard errors.

**Figure S2** The histogram of the residuals from Model 1

**Figure S3** The plot of the residuals against fitted values from Model 1.

**Figure S4** The histogram of the residuals from Model 2.

**Figure S5** The residuals and fitted values from Model 2 (which included a single, binomial fixed effect).
